# Deep learning for early detection of Zenker’s diverticulum based on swallowing sound analysis

**DOI:** 10.1007/s11548-026-03727-8

**Published:** 2026-06-03

**Authors:** Daniel Ostler-Mildner, Alissa Jell, Matthias Seibold, Hubertus Feussner, Simone Graf, Dirk Wilhelm, Jonas Fuchtmann

**Affiliations:** 1 Research Group MITI, TUM University Hospital, School of Medicine and Health, Technical University of Munich, Munich, Germany; 2 Department of Surgery, TUM University Hospital, School of Medicine and Health, Technical University of Munich, Munich, Germany; 3 Research in Orthopedic Computer Science, University Hospital Balgrist, Zurich, Switzerland; 4 Innsbruck Medical University, Clinic for Hearing-, Speech- and Voice Disorders, Innsbruck, Austria

**Keywords:** Computer-assisted diagnostics, Biomedical acoustics, Deep learning, Dysphagia, Cervical auscultation

## Abstract

**Purpose:**

Patients with Zenker’s diverticulum (ZD) often suffer from oropharyngeal dysphagia that can go undiagnosed for years. Diagnosis of ZD typically requires specialized centers and videofluoroscopy. Our study aims to create a noninvasive, accessible, sound-based screening tool for healthcare professionals to reduce diagnostic barriers and enable earlier detection of ZD.

**Methods:**

We developed a two-stage deep learning model to detect ZD using cervical auscultation sounds. The first stage identifies swallowing sounds (idle vs. swallow), and the second classifies detected swallows as healthy or pathological (Healthy vs. ZD). We used transfer learning with a pre-trained audio spectrogram transformer (AST) backbone and fine-tuned it for our task. A fivefold cross-validation protocol was applied to evaluate the model’s performance. For data collection, we built a portable cervical auscultation device to gather recordings from 23 ZD patients and 27 healthy volunteers.

**Results:**

The proposed method achieved a patient-level ZD diagnosis accuracy of $88.7 \pm 7.7 \%$ and an F1-score of $87.6 \pm 8.3 \%$. We report the intermediate results for the individual stage on a snippet level and perform an ablation study to justify our design decisions and benchmark our approach.

**Conclusion:**

This study demonstrates, to our knowledge, the first deep learning-based cervical auscultation approach for identifying ZD. The results indicate that auscultation-driven AST-based models can provide clinically meaningful sensitivity and may help to lower diagnostic barriers, enable earlier referral, and ultimately reduce healthcare costs in dysphagia care.

## Introduction

Dysphagia can impact one of the most essential human needs: the consumption of liquids and food. It is defined as a disturbance in the intake or transport of food to the stomach. Since swallowing is a complex process that requires the precise coordination of multiple muscles and neural signals, disruptions can occur due to structural, functional, or neurological lesions. One such structural disorder is Zenker’s diverticulum (ZD), also known as a pharyngeal pouch, which involves an anatomical alteration at the entrance of the esophagus. It is characterized by an out-pouching of tissue in the Killian triangle caused by a dysfunction of the cricopharyngeal muscle [1].

ZD patients suffer from a range of symptoms, including dysphagia, globus pharyngis, regurgitation, odynophagia, halitosis, aspiration, frequent pulmonary infections, inability to intake oral medicine, malnutrition, and even social isolation. ZD is a rare disease predominantly affecting the aged population, with an estimated prevalence of approximately 3 out of 100,000 patients [2]. Symptoms may persist for extended periods, from weeks to years, before ZD is formally diagnosed [1]. The etiologic workup of dysphagia, particularly the identification of rare structural causes such as ZD, is a complex process that requires highly qualified medical staff and specialized, advanced instrumental techniques, both of which are only available at specialized centers. Commonly, video endoscopy, i.e., fiber-optic endoscopic evaluation of swallowing (FEES), is performed in a first ear, nose, and throat (ENT) examination for the diagnosis of dysphagia, with a subsequent videofluoroscopic swallowing study (VFSS) in patients with suspected ZD [3]. While VFSS is the gold standard in the diagnosis of ZD, dynamic X-ray is not widely available, especially in smaller medical centers, and thus underperformed in patients with dysphagia. Furthermore, radiation exposure makes frequent testing inappropriate [4].

Recent technological advancements, particularly in the field of machine learning (ML), have shown promising improvements in dysphagia diagnostics. For instance, [5] applied an ML-based approach for audio feature selection for relevant biomarkers (e.g., frequency, energy, or spectral descriptors like pitch, loudness, and MFCCs) for detecting dysphagia in voice data. Furthermore, the detection of dysphagia based on voice data with a spectrogram-based approach has been demonstrated by [6]. Moreover, [7] showcased the feasibility of post-stroke dysphagia detection using mel spectrograms derived from vocalization data, achieving participant-level sensitivity of 89% and specificity of 79%.

While recent studies have successfully applied machine learning to *voice audio* for dysphagia detection, we argue that swallowing sounds represent a more direct signal for detecting structural pharyngeal pathologies like ZD. Voice audio primarily reflects vocal fold and laryngeal function, capturing downstream consequences of dysphagia rather than underlying structural dysfunction. In contrast, swallowing sounds directly encode pharyngeal and esophageal dynamics, providing acoustic biomarkers aligned with structural disorders like ZD. This physiological distinction motivates our focus on cervical auscultation, and aligns with the suggested methodology of Khalifa et al. [8] using high-resolution cervical auscultation (HRCA) for general dysphagia assessment but not yet systematically applied to ZD detection.

To our knowledge, this is the first study to apply machine learning-based cervical auscultation specifically for ZD detection. While previous methods used classical handcrafted feature-based methods or mel spectrograms combined with CNNs, we apply a state-of-the-art transformer-based architecture to solve the problem. Furthermore, we propose a custom capture hardware setup designed for reproducible, portable, and bedside-compatible bilateral acquisition of cervical swallowing sounds.

By targeting the acoustic phenomena associated with ZD pathophysiology, we develop a proof-of-concept screening approach that may lower the initial diagnostic barriers and thus promote early detection of ZD. We present three key contributions:

- A custom recording platform enabling placement-robust setup for high-quality capturing of swallowing sounds, tailored for bedside use.
- A two-stage state-of-the-art transformer-based pipeline for detecting ZD
- A clinically grounded and thorough validation on a cohort of 23 ZD patients and 27 healthy subjects

## Methods

### Hardware setup

**Fig. 1:**
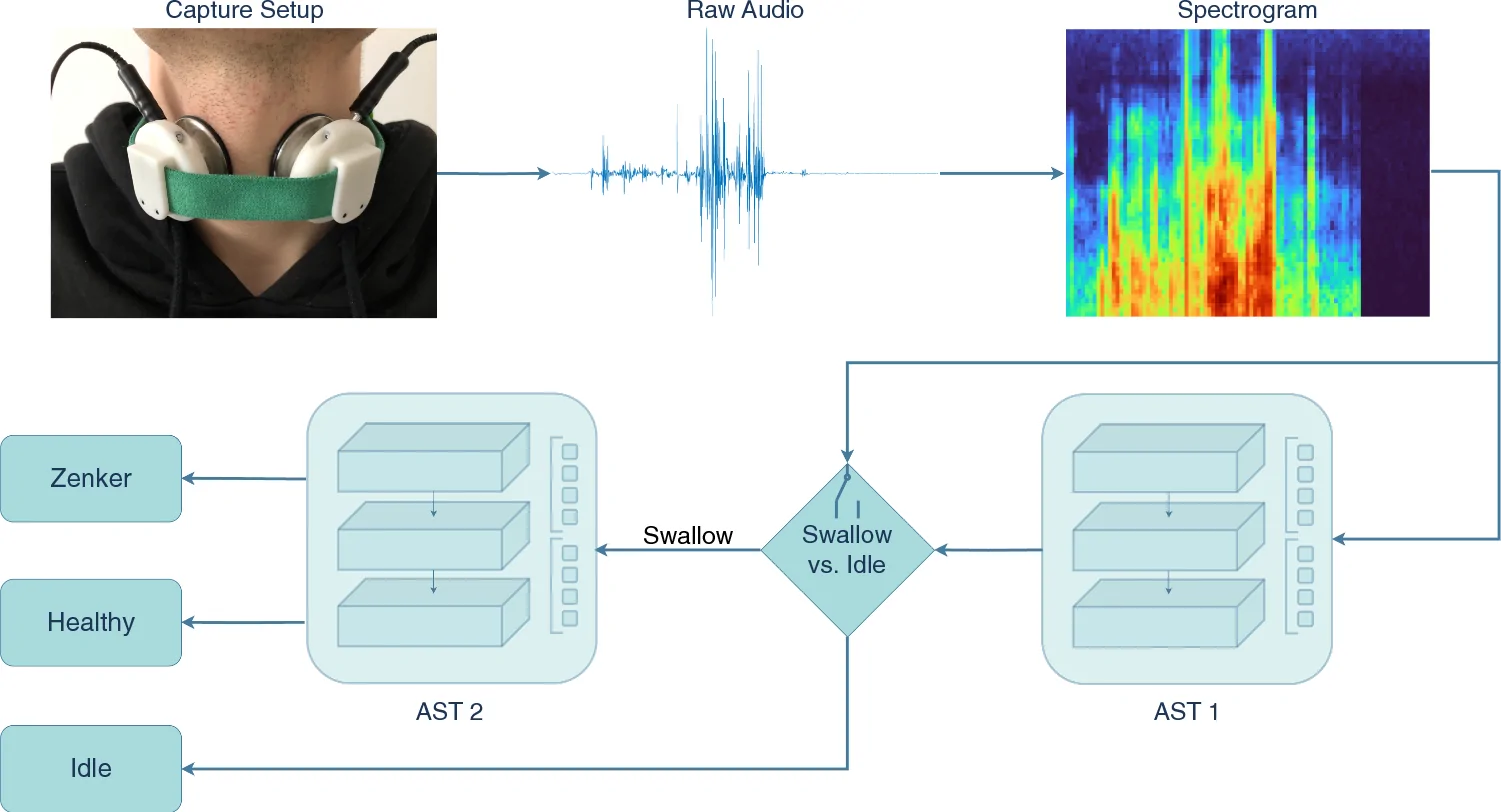
Signal processing workflow for classification: Audio signals from cervical auscultation are captured by microphone-equipped stethoscopes, recorded, and converted from a waveform into a log-mel spectrogram. These spectrograms serve as input for the first AST stage, distinguishing between “swallow” and “idle” sounds. If a swallowing sound is predicted, the same input spectrogram is used for the second stage of AST, which classifies between healthy and potential ZD

To support reproducible bilateral acquisition of cervical swallowing sounds in a portable setup, we developed a custom capture system with stable neck placement. A flexible collar, adjustable to fit individual anatomical variations, is used to secure the recording apparatus in place, while minimizing the risk of choking or discomfort for the volunteer during the data-gathering process. We incorporate two medical-grade stethoscope heads (3 M Littmann Classic III, 3 M, Saint Paul, USA) and miniature microphones (4660-OC-H-B00, DPA MICROPHONES A/S, Kokkedal, Denmark) to record cervical sounds simultaneously from both sides of the neck. During the training data acquisition phase, we use a handheld recorder (H5, ZOOM Corporation, Tokyo, Japan), enabling bedside audio acquisition at a sample rate of 48 kHz and 32 bit. Each recording yields two tracks: one for the left and one for the right stethoscope. The top left part of Fig. 1 shows the custom microphone collar developed within this work.

### Data collection

After gaining approval from the local ethics committee at Technical University Munich (TUM) (#658/21 S), we initiated the process of collecting sample data for audio-based detection of dysphagia. Data sampling involved patients who were referred for extended dysphagia examinations to the outpatient clinic at the Department of Otorhinolaryngology or the Upper GI Functional Laboratory at the TUM University Hospital Rechts der Isar in Munich. Patients with confirmed ZD (based on FEES or VFSS diagnostics) were included in the study, resulting in a collective of 23 ZD patients (age: mean (SD) 68.1 (12.2), min.–max.: 31–83, male: 16 (69.6%), female: 7 (30.4%)).

Furthermore, we included 27 healthy volunteers as a control group (age: mean (SD) 38.7 (15.5), min.–max.: 21–69, male: 16 (59.3%), female: 11 (40.7%)). The volunteers were examined by medical experts and included in the healthy control group after they were considered healthy according to internationally established metrics for the classification of dysphagia, i.e. an Eating Assessment Tool-10 (EAT-10) [9] with a participant’s rating below 3. Informed consent was obtained from all participants.

For each study subject, recordings of swallowing sounds were made according to a defined protocol of at least five consecutive dry swallows (i.e., only saliva) and at least five individual wet (i.e., water) swallows.

Although the recordings were obtained in different environments (outpatient clinic vs. functional laboratory), we ensured that the recording conditions were as similar as possible. The subjects were seated in an upright position in a quiet room, and the microphone collar was placed around their necks. The recordings were supervised by medical experts to ensure correct execution of the swallowing protocol.

### Data analytics

#### Audio preprocessing

The swallowing sounds were segmented into one-second snippets by medical experts using time code markers during manual preprocessing and labeling that was performed using Audacity (v3.6). This was performed in a multi-step process. Initially all recordings were assessed by medical students who identified the individual swallowing events and labeled them in a first pass. Subsequently, an experienced physician reviewed the annotations and made small adjustments wherever necessary. The time code markers ensure consistent audio for the same swallowing event across both left and right stethoscope channels. Based on the subject’s diagnosis, all corresponding files are labeled as either *healthy* or *zenker*. Additionally, audio between swallowing events and other non-swallowing events (e.g., coughing, throat clearing, speech) are labeled and exported in contiguous segments with durations ≥ 1 s. For simplicity, all non-swallowing sounds are grouped as *idle*. This approach enables the classification of entire sequences of diagnostic recordings as described in Section “Ablation study.”

Expert annotations defined the swallowing events and class labels with a target duration of 1.00 s. To address minor duration deviations in the manually selected time windows, we applied an automated post-processing Python script to all annotated snippets. If needed, signals were resampled to 48 kHz and length-normalized to exactly 48 000 samples.

For swallowing sounds, length normalization was applied only if the annotated segment deviated from the target duration. In these cases, a common 1.00 s window was selected for each L/R pair, preserving temporal inter-channel alignment. Recordings shorter than 1.00 s were zero-padded symmetrically; recordings between 1.00 s and 1.50 s were trimmed to be centered around the highest short-time root mean square (RMS) energy region. No curated swallowing segment exceeded 1.50 s. In the curated pre-normalization swallow set, 81.4 % of snippets already had the exact target length, and only 0.56 % deviated by more than 10 ms. Thus, this post-processing step adjusted only minor duration deviations in the manually selected time windows rather than redefining the annotated swallowing events.

The RMS-based centering step was only applied to swallowing segments. Idle labels represent longer non-swallowing intervals between swallowing events. Hence, these sections were split into consecutive 1.00 s snippets without RMS-based centering. The remaining last snippet of an idle section was zero-padded to 1.00 s if the original signal was at least 0.75 s long, or discarded otherwise. The final one-second audio outputs were written as 24-bit PCM WAV.

In total, the collected dataset consists of 698 one-second snippets from Zenker patients, 734 snippets from healthy individuals, and 2819 idle snippets. It is to be noted that each side of the neck (left and right channels) is treated as a separate sample at the model input stage. This data capture protocol increases the number of channel-level training observations, since one swallow event will result in two similar yet not equal sound samples. This can help improve the robustness and generalization capabilities of our machine learning models. Moreover, ZD can occur on either side of the esophagus, and the sound propagation can vary depending on the exact location of the pouch. By analyzing both channels separately, we increase the likelihood of capturing the relevant acoustic signatures regardless of the location of the diverticulum. This approach is particularly beneficial for future diagnostic applications in new patients. Given the proof-of-concept nature of this study and the limited dataset size, we do not yet explicitly exploit relational information between the left and right channels at the model level; modeling inter-channel relationships, such as lateral asymmetry or timing differences, remains future work on larger cohorts. All files are associated with a unique subject ID, ensuring patient-level isolation between training, validation and test sets and preventing data leakage across folds during cross-validation. By applying fivefold cross-validation, the dataset is split into training (80%) and test/hold-out set (20%) at the subject level. Within the training set of each fold, a further split of 25% for the validation set is performed, again at the subject level. This ensures that the model is validated during training and tested after training using entirely unseen data during training, respectively.

#### Detection architecture

The proposed method is designed as a two-stage approach, as depicted in Fig. 1. In the first stage, a fine-tuned AST model is used to distinguish between *swallowing* sounds and *idle* (non-swallowing) sounds. In the second stage, a second fine-tuned AST model is applied to the sounds identified as swallowing by the first stage to further classify them into two categories: *healthy* and pathological *Zenker* swallowing sounds.

**Model** ∣ We employ a Audio Spectrogram Transformer (AST) model architecture that operates on log-mel spectrograms, which are image-based frequency–time representations of the raw audio waveforms and provide a compact but information-preserving representation of the acoustic signals. The spectrograms are divided into non-overlapping patches, which are linearly embedded and passed through a stack of transformer layers, allowing the model to capture long-range dependencies across both time and frequency.

In our study, we utilize the fine-tuned version *ast-finetuned-audioset-10–10−0.4593* pre-trained on the large-scale AudioSet corpus.^1^ This model serves as the backbone for both classification stages, fine-tuning it for our specific tasks of distinguishing between swallowing and idle sounds, and between healthy and Zenker swallowing sounds.

**Training and data augmentation** | We employed fivefold subject-level cross-validation with per-fold normalization. We tested data augmentation during training: Gaussian noise (SNR: 10 -20 dB), gain scaling (–6–6 dB), temporal stretching (rate 0.8 −1.2), pitch shifting (–4 to 4 semitones), and time masking (1–20%), applied with probability 0.8. To address class imbalance, we evaluated cross-entropy and focal loss; additionally, we evaluated label smoothing as a calibration-oriented regularizer. The final stage-specific training configuration is determined by the ablation study and end-to-end patient-level validation (3.3). The training was performed on an System with a NVIDIA RTX 4000 SFF Ada GPU, running Ubuntu 22.04 LTS with Python 3.10.12, PyTorch 2.7.1, and Hugging Face Transformers 4.52.4. The code has been made publicly available to disclose all further implementation details.

**Fig. 2:**
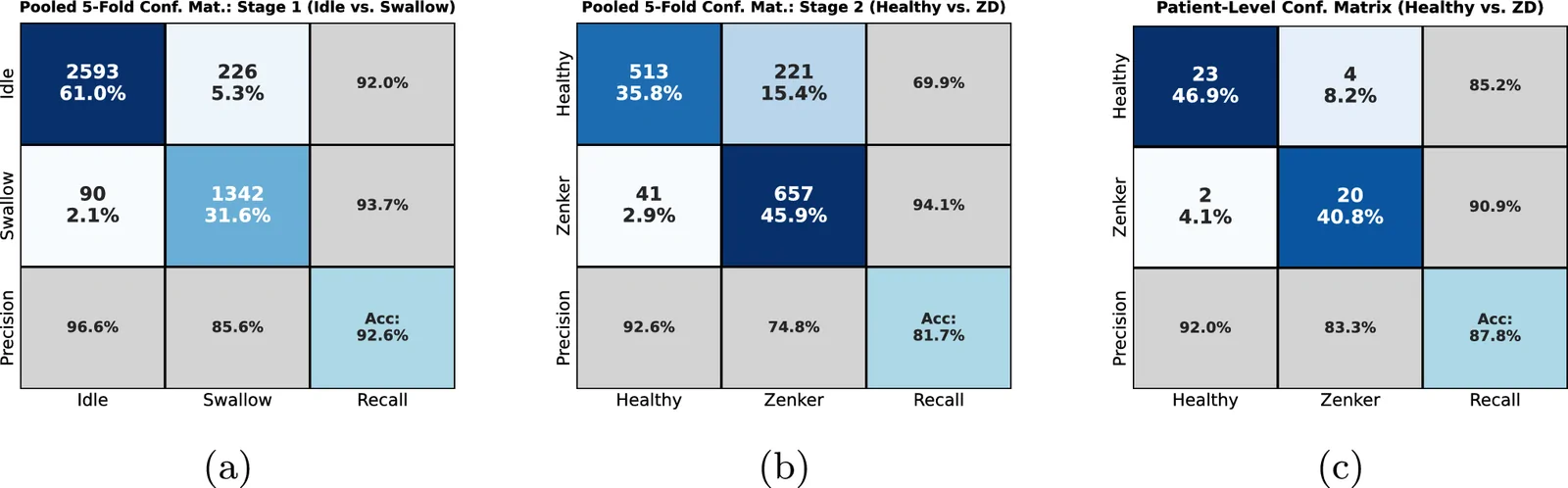
Confusion matrices for both binary classification tasks. **a** Pooled confusion matrix for swallowing vs. idle classification by the final AST models for stage 1. **b** Pooled confusion matrix for ZD vs. healthy classification by the final AST models for stage 2. **c** Confusion matrix for the patient-level classification based on stage 1 + stage 2 (at $\text {ZSR}>0.7$). Please note that we report the pooled (micro) accuracy here, rather than the macro accuracy reported in the remainder of the manuscript

## Results

In the following sections, we first report the individual performance of stage 1 (idle vs. swallow detection) in Section “Stage 1 results—idle vs. swallow (snippet level)” and stage 2 (healthy vs. ZD detection) in Section “Stage 2 results—healthy vs. Zenker (snippet level).” We perform an ablation study to justify the reported model selection (ablation variant A1b for both stages) in Section “Ablation study” and evaluate the fully assembled pipeline on full recordings of individual patients in Section “Patient-level classification,” where the proposed method achieved an overall F1 score of $87.6 \pm 6.1 \%$. The detailed results for these main results are given in Table 1 and the confusion matrix in Fig. 2. Finally, we compare the method with a single-stage variant in Section “Single-stage comparison.”

### Stage 1 results—idle vs. swallow (snippet level)

As depicted in Fig. 1, the first classification stage of the detection pipeline is the classification between swallowing and idle sounds. The idle class encompasses any period in the audio where swallowing is absent, including sounds related to breathing, coughing, speaking, and other non-swallowing activities. The dataset used in this stage consists of 2819 idle snippets and 1432 (698 ZD + 734 healthy) swallow snippets resulting in an imbalanced dataset.

**Fig. 3:**
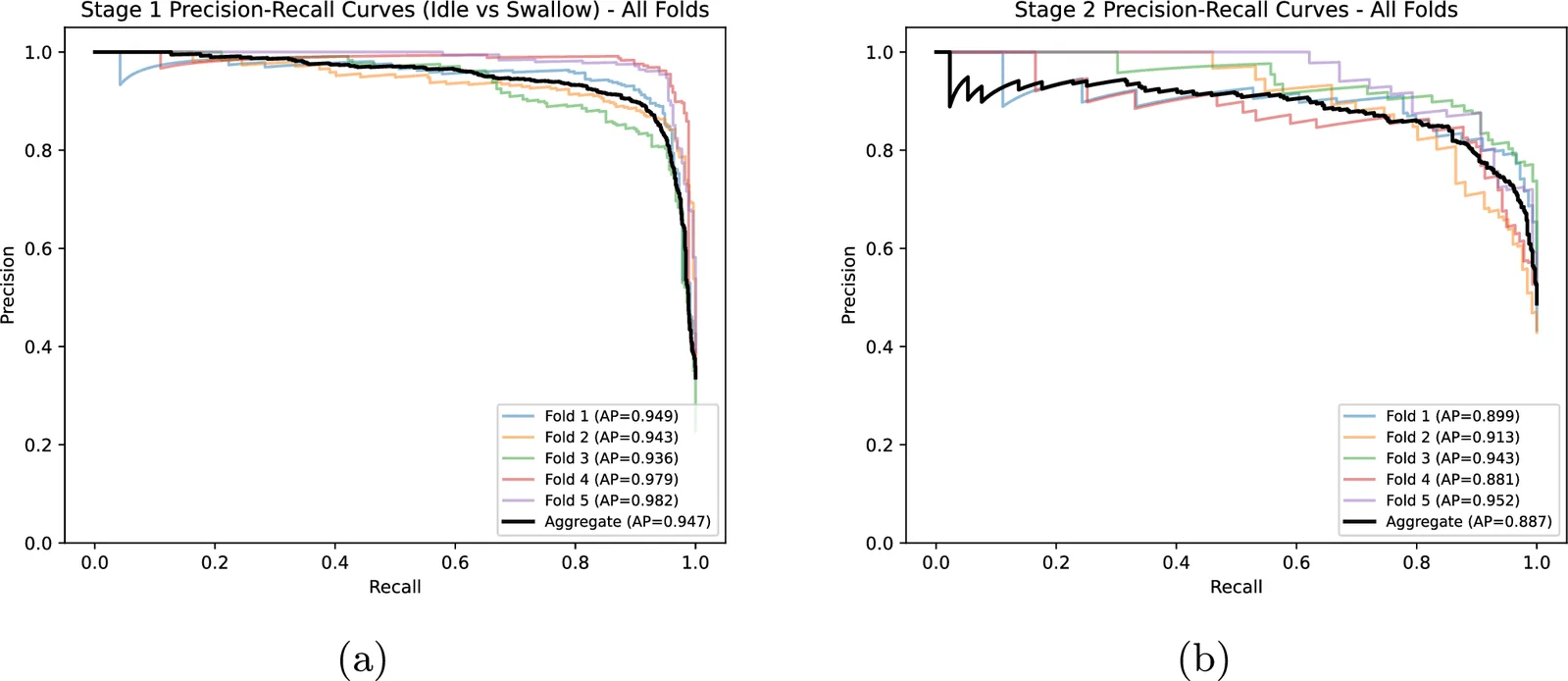
Precision–recall curves for stage 1 (**a**) and stage 2 (**b**) across all five folds, showing the trade-off between precision and recall across different thresholds. The mean curve across all folds is shown in bold black, and AUPRC values are reported in the legend

Table 1 presents the performance metrics, including accuracy, precision, recall, specificity, and F1 score for this classification task. The area under the curve (AUC) in the precision–recall curves (Fig. 3a) confirm the model’s effectiveness in identifying swallowing events within the cervical auscultation recordings. The corresponding confusion matrix aggregating predictions across all test sets is shown in Fig. 2a. In addition to this default configuration, we evaluate simpler baselines and ablations of augmentation, focal loss, and label smoothing; results are summarized in 3.3.

**Table 1:** Cross-validation performance summary (mean±SD)

	Macro acc.	Prec.	Rec.	Spec.	F1
Stage 1	0.914±0.036	0.845±0.133	0.926±0.049	0.902±0.105	0.876±0.061
Stage 2	0.736±0.046	0.675±0.108	0.894±0.069	0.579±0.144	0.762±0.051
Patient	0.887±0.077	0.867±0.183	0.920±0.183	0.853±0.202	0.876±0.083

The ablation variant A1b was selected as stage 1 and stage 2; patient-level classification at operating point ZSR   >0.70; Macro Acc.: macro accuracy; Prec.: precision; Rec.: recall/sensitivity; Spec.: specificity

### Stage 2 results—healthy vs. Zenker (snippet level)

The differentiation between healthy swallowing sounds and those associated with ZD, is performed in the second stage. The results across the five folds are presented in Table 1. The corresponding confusion matrix aggregating predictions across all test sets for stage 2 is shown in Fig. 2b. The precision–recall curves for this classification task are depicted in Fig. 3b.

**Table 2:** Snippet-level cross-validation results (mean±SD across 5 folds)

Variant	Macro acc.	Prec.	Rec.	Spec.	F1
Stage 1 (idle vs. swallow)—snippet level					
SE-ResNet (baseline)	0.907±0.020	0.875±0.063	0.880±0.044	${\textbf {0.934}}\pm {\textbf {0.036}}$	0.876±0.037
A0 (CE, no aug/LS/focal)	0.914±0.014	$\mathbf {0.878\pm 0.120}$	0.895±0.054	$\mathbf {0.934\pm 0.063}$	0.879±0.042
A1 (+aug)	0.918±0.020	0.863±0.127	0.913±0.050	0.923±0.070	0.880±0.054
A1b (+focal)	$\mathbf {0.933\pm 0.016}$	0.872±0.115	$\mathbf {0.937\pm 0.031}$	0.929±0.058	$\mathbf {0.898\pm 0.053}$
A2 (+aug+focal)	0.909±0.030	0.832±0.161	0.922±0.073	0.895±0.105	0.862±0.072
A3 (+aug+focal+LS)	0.914±0.036	0.845±0.133	0.926±0.049	0.902±0.105	0.876±0.061
Stage 2 (healthy vs. Zenker)—snippet level					
SE-ResNet (baseline)	0.682±0.065	0.616±0.086	0.936±0.042	0.427±0.166	0.739±0.052
A0 (CE, no aug/LS/focal)	$\mathbf {0.819\pm 0.067}$	$\mathbf {0.774\pm 0.123}$	0.917±0.061	0.722±0.189	0.831±0.063
A1 (+aug)	0.763±0.062	0.758±0.121	0.797±0.120	0.730±0.185	0.764±0.056
A1b (+focal)	$\mathbf {0.819\pm 0.031}$	0.752±0.057	$\mathbf {0.940\pm 0.046}$	0.698±0.086	$\mathbf {0.833\pm 0.028}$
A2 (+aug+focal)	0.742±0.043	0.751±0.102	0.751±0.187	$\mathbf {0.733\pm 0.196}$	0.732±0.061
A3 (+aug+focal+LS)	0.736±0.046	0.675±0.108	0.894±0.069	0.579±0.144	0.762±0.051

Macro Acc.=macro accuracy; Prec.=precision; Rec.=recall/sensitivity; Spec.=specificity; CE=cross-entropy loss; focal=focal loss; aug=augmentations; LS=label smoothing, using the following parameters: focal γ 2.0, LS 0.07 stage 1 /0.09 stage 2

Bold indicates the best mean value per metric within each stage; ties in mean are both shown in bold

### Ablation study

Ablations are reported to quantify the contribution of augmentation, focal loss, and label smoothing, and are summarized in Table 2. Based on their performance metrics, the fine-tuned model variants A1b, i.e., AST with focal loss (focal γ=2.0) were selected as the final models for both stage 1 and stage 2.

**Fig. 4:**
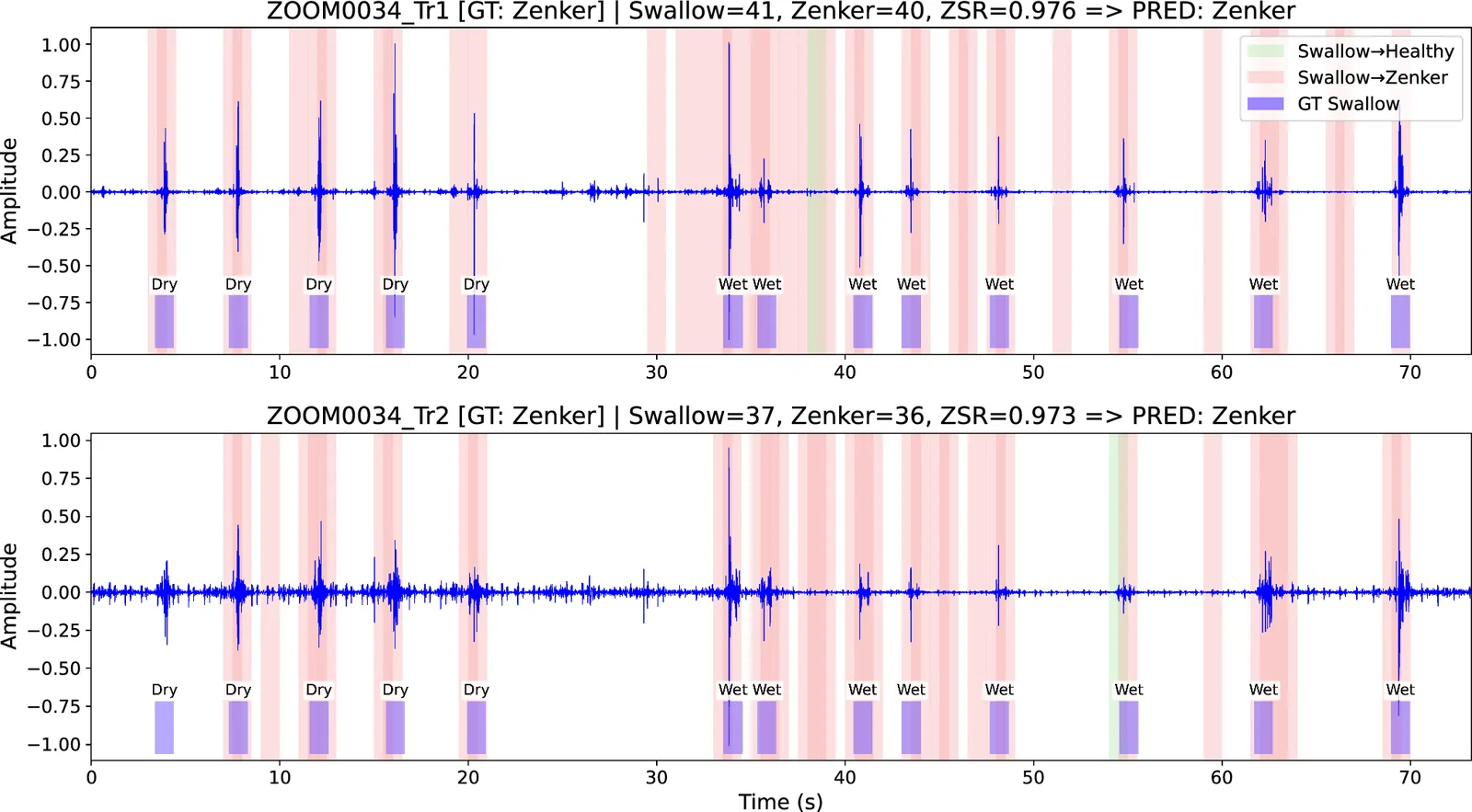
Results on two-stage patient-level analysis for a patient with ZD. The two rows display the waveform for the left and right tracks. The colored overlays indicate the predicted classes for each window (green: healthy, red: ZD); swallowing ground truth is indicated as purple. The Zenker-to-swallow ratio (ZSR) is calculated for each track and averaged for the final patient-level prediction with $t_{ZSR}=0.7$

### Patient-level classification

As depicted in the flow diagram in Fig. 1, we implemented a two-stage approach, combining the two above-described binary classifiers. In stage 1, the model differentiates between swallowing and idle sounds. If a swallowing sound is detected, stage 2 further classifies it as either healthy or ZD.

Because the clinical endpoint is patient-level screening, we evaluate the final pipeline combination (stage 1 A1b + stage 2 A1b) in terms of real-world applicability. For this purpose, we presented the original uncut audio recordings from subjects collected during their examinations which formed the basis for the snippets. We ensured that none of the audio content was previously used in training or validation for either the stage 1 or stage 2 classifier. The classification was run on both tracks (from the left and right stethoscopes) with a sliding-window technique using 1-second windows with a 0.5-sec hop size.

In Stage 1, windows with predicted swallow probability above threshold $t_1\ge 0.5$ were classified as swallowing sounds and passed to Stage 2. In Stage 2, swallow windows with predicted Zenker probability above threshold $t_2\ge 0.5$ were classified as ZD, otherwise as healthy.

**Fig. 5:**
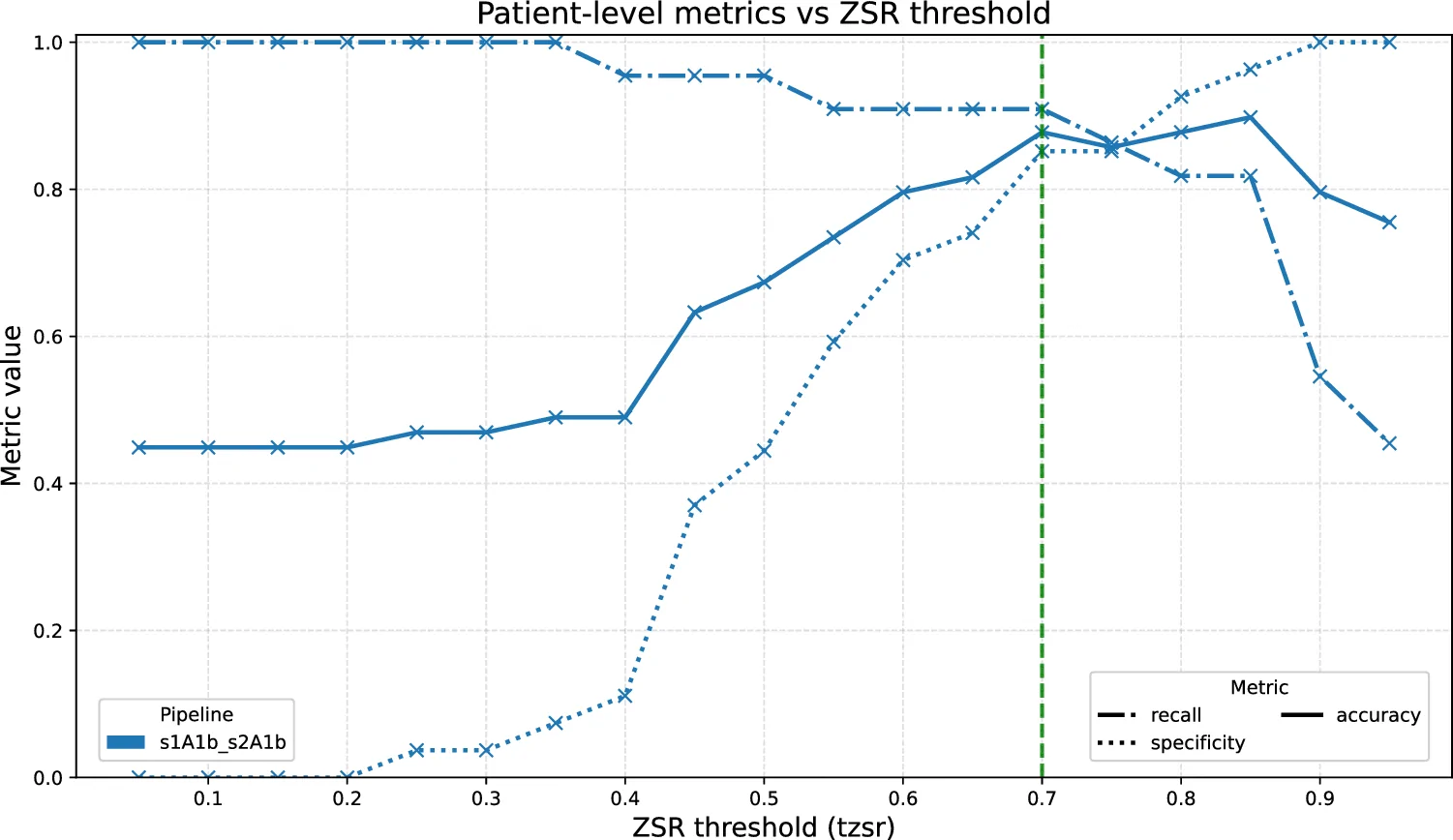
Patient-level operating point selection via ZSR threshold sweep. Patient-level sensitivity (recall), specificity, and accuracy as a function of the ZSR threshold $t_{\textrm{ZSR}}$ for the two-stage pipeline (A1b+A1b) on held-out, uncut recordings. We select $t_{ZSR}=0.7$ as an operating point (green line). All values are reported as mean over all cv folds as reported in Table 1

For each audio track, we calculated the Zenker-to-swallow ratio (ZSR), defined as the number of detected Zenker windows divided by the total number of detected swallowing windows. The ZSR values from both stethoscope tracks (left and right) were averaged to obtain a single patient-level ZSR. To select the patient-level decision threshold $t_{ZSR}$, we performed a sweep over candidate values and computed patient-level sensitivity (recall), specificity, and accuracy on the held-out long recordings using the fixed window-level thresholds $t_1=t_2=0.5$. As shown in Fig. 5, $t_{ZSR}=0.7$ provides a favorable operating point near the knee of the sensitivity–specificity trade-off Therefore, we set $t_{ZSR}=0.7$ for patient-level reporting and classify a subject as ZD if $\text {ZSR} \ge 0.7$, otherwise as healthy.

The patient-level analysis was performed on 27 healthy subjects and 22 ZD patients (one patient was excluded due to a missing audio recording of one side). We did not apply any further post-processing or smoothing of the classification results.

The detailed results for patient-level inference across 49 patients are given in Table 1. Figure 2c illustrates the confusion matrix for patient-level inference. Figure 4 visualizes the results for the patient-level classification for one subject diagnosed with ZD.

### Single-stage comparison

Finally, we justify the two-stage approach by comparing our method with a single-stage implementation that employs the same AST model but trained in a multi-class fashion and evaluated over all 49 individual recordings identical to the process described in Section “Patient-level classification.” The model achieves a macro accuracy of 64.4%, precision, recall, and specificity of 53.8%, 95.5%, and 33.3%, respectively, as well as a F1 score of 68.9%. These results support the implementation of our proposed two-stage pipeline.

## Discussion

Our results indicate that our approach has great potential to effectively distinguish between healthy swallowing sounds and those associated with ZD. High values for the AUC metrics of the precision–recall curves suggest that AST can capture subtle acoustic differences associated with ZD, thereby meeting our objectives of high accuracy and clinical scalability.

Compared to VFSS, the current gold standard, our cervical auscultation approach offers several advantages: it is noninvasive, radiation-free, uses a portable recording device, and can be performed by non-specialist healthcare providers (HCPs) in various clinical settings. This accessibility could enable earlier screening and referral, particularly in primary care or smaller medical centers where advanced imaging is unavailable.

The notable performance difference between Stage 1 (idle vs. swallow, AUPRC: 0.947) and Stage 2 (healthy vs. Zenker, AUPRC: 0.887) indicates the inherent complexity of this task. Stage 1 distinguishes swallowing from idle periods containing breathing, coughing, throat clearing, and speech. Despite high-energy events in the idle class, swallowing exhibits distinctive temporal–spectral patterns enabling reliable identification, as evidenced by the confusion matrix in Fig. 2a showing strong diagonal performance with minimal misclassification (93.7% swallow recall, 92.0% idle specificity). Stage 2, however, must differentiate between healthy and pathological swallows that are acoustically similar, which requires the detection of subtle pathological features. This task is rendered more difficult by the limited sample size. The Stage 2 confusion matrix (Fig. 2b) reveals asymmetric misclassification between classes, with high ZD recall (94.1%), and reduced healthy specificity (69.9%), indicating the increased difficulty of this fine-grained discrimination task.

The small dataset (23 ZD patients, 27 healthy volunteers) particularly impacts Stage 2 performance. Limited training samples constrain learning the full spectrum of acoustic variability, with individual differences in anatomy, swallowing technique, and heterogeneous ZD presentation potentially underrepresented. The age distribution is imbalanced (ZD 68.1 vs. healthy 38.7 years). To probe confounding, we performed age-overlap stress tests where age-only AUROC decreased (e.g., age ranges 31–69: 0.622; 50–69: 0.414) while the model’s AUROC score remained high (0.94 and 0.93, respectively).

Additionally, the model currently does not account for varying severity of the ZD. A correlation analysis with variables such as diverticulum size and location, as well as swallowed bolus sizes or consistencies and their impact on acoustic characteristics, could provide further insights and refine the detection accuracy. Potential biases related to demographic or clinical characteristics within the dataset should also be examined. The goal of our study was the development of a novel method for the diagnosis of ZD. The robustness of the proposed methodology to distinguish ZD from other types of dysphagia should be evaluated in future work.

Future work must prioritize multicenter expansion with substantially larger, age-matched ZD patient and control cohorts to address these fundamental limitations.

While this study primarily establishes a proof of concept for ZD detection from these recordings, we note plausible deployment paths such as (i) on-device inference on GPU-enabled edge hardware (e.g., NVIDIA Jetson) or (ii) a client–server workflow with remote inference. Fully real-time processing is not clinically required and results within minutes after acquisition would suffice for screening. However, the deployment engineering is beyond the scope of this work and all inference experiments were run on a standard GPU-equipped PC. On a 73.2 s dual-channel recording, end-to-end inference required 39.2 s, with a peak GPU memory allocation of approximately 6.7 GB, providing an estimate of the memory footprint in our current implementation.

## Conclusion

This study addresses the critical challenge of early and accessible diagnosis of Zenker’s diverticulum (ZD). In clinical practice, symptoms may persist for weeks to years before a definitive diagnosis is established, and diagnostic confirmation typically requires specialized diagnostic centers that rely heavily on expert knowledge and resource-intensive methods such as videofluoroscopy [1]. To overcome these barriers, our project aims to democratize access to diagnosis for the small but significant patient population affected by oropharyngeal dysphagia. We propose an audio analysis-based diagnostic tool that utilizes noninvasive cervical auscultation of swallowing sounds for classification via a deep learning pipeline. Although still a work in progress, we have successfully identified swallowing events and demonstrated the potential to detect pathological conditions associated with ZD. This method could be particularly beneficial in non-specialized settings or regions with limited access to advanced medical infrastructure, enabling a wider range of HCPs to potentially detect ZD. Moving forward, expanding our dataset and integrating this tool into portable, near-real-time diagnostic systems could advance dysphagia screening, especially in resource-limited settings. This could ultimately improve patient outcomes and reduce healthcare costs.

## Data Availability

The datasets generated and analyzed during the current study cannot be publicly shared due to privacy concerns and restrictions imposed by the ethical approval. Anonymized data may be made available from the corresponding author upon reasonable request and subject to appropriate data use agreements. The code for data processing, model training, and evaluation is available at https://github.com/daostler-tum/zenker-audio-detection.git.

## Footnotes

1 AudioSet: https://research.google.com/audioset/index.html.
